# Inhibition of RIPK1 or RIPK3 kinase activity post ischemia-reperfusion reduces the development of chronic kidney injury

**DOI:** 10.1042/BCJ20240569

**Published:** 2025-01-22

**Authors:** Aspasia Pefanis, Anjan K. Bongoni, Jennifer L. McRae, Evelyn J. Salvaris, Nella Fisicaro, James M. Murphy, Francesco L. Ierino, Peter J. Cowan

**Affiliations:** 1 Immunology Research Centre, St Vincent’s Hospital, Melbourne, Victoria, Australia; 2 Department of Medicine, The University of Melbourne, Melbourne, Victoria, Australia; 3 Department of Nephrology, St Vincent’s Hospital, Melbourne, Victoria, Australia; 4 Walter and Eliza Hall Institute of Medical Research, Parkville, Melbourne, Australia; 5 Department of Medical Biology, The University of Melbourne, Melbourne, Victoria, Australia; 6 Drug Discovery Biology, Monash Institute of Pharmaceutical Sciences, Monash University, Parkville, Victoria, Australia

**Keywords:** acute kidney injury, ischemia-reperfusion, kidney fibrosis, kinase inhibitor injury, programmed necrosis

## Abstract

Ischemia-reperfusion injury (IRI) occurs when the blood supply to an organ is temporarily reduced and then restored. Kidney IRI is a form of acute kidney injury (AKI), which often progresses to kidney fibrosis. Necroptosis is a regulated necrosis pathway that has been implicated in kidney IRI. Necroptotic cell death involves the recruitment of the RIPK1 and RIPK3 kinases and the activation of the terminal effector, the mixed lineage kinase domain-like (MLKL) pseudokinase. Phosphorylated MLKL causes cell death by plasma membrane rupture, driving ‘necroinflammation’. Owing to their apical role in the pathway, RIPK1 and RIPK3 have been implicated in the development of kidney fibrosis. Here, we used a mouse model of unilateral kidney IRI to assess whether the inhibition of RIPK1 or RIPK3 kinase activity reduces AKI and the progression to kidney fibrosis. Mice treated with the RIPK1 inhibitor Nec-1s, either before or after IR, showed reduced kidney injury at 24 hr compared with controls, whereas no protection was offered by the RIPK3 inhibitor GSK´872. In contrast, treatment with either inhibitor from days 3 to 9 post-IR reduced the degree of kidney fibrosis at day 28. These findings further support the role of necroptosis in IRI and provide important validation for the contribution of both RIPK1 and RIPK3 catalytic activities in the progression of kidney fibrosis. Targeting the necroptosis pathway could be a promising therapeutic strategy to mitigate kidney disease following IR.

## Introduction

The kidneys are particularly susceptible to ischemia-reperfusion injury (IRI), which occurs after the blood supply to the kidney is temporarily interrupted [1]. During ischemia, lack of oxygen and nutrients results in a reduction in oxidative metabolism and accumulation of metabolic waste products. There is a switch from aerobic to anaerobic metabolism with resultant acidosis causing cellular oedema and cell death [2–4]. Reperfusion returns the kidney to aerobic metabolism with pH normalisation [5–7] but also generates reactive oxygen species, which together with cytokine production and complement activation cause inflammation [8,9], microvascular dysfunction [10–13] and immune activation [14–16]. This, in turn, damages functional cellular components with structural impairment and cell death causing acute kidney injury (AKI). AKI often progresses to kidney fibrosis [17,18], causing significant patient morbidity and mortality with escalating healthcare costs [19,20]. There are no effective clinical therapies to prevent AKI or the subsequent progression to kidney fibrosis following IR.

There is a growing body of literature supporting the involvement of necroptosis in kidney IRI [21]. The necroptosis pathway relies on three core effector proteins: receptor interacting protein kinases 1 and 3 (RIPK1 and RIPK3) and the terminal pathway effector pseudokinase, mixed lineage kinase domain-like (MLKL). The best-studied stimulus for the activation of necroptosis is Tumor Necrosis Factor (TNF); however, necroptosis can also be initiated by other members of the TNF death ligand family, interferons, TLR signalling and viral infection [22]. In scenarios where the pro-NF-κB activity of the cIAP E3 ubiquitin ligase family and the pro-apoptotic function of Caspase-8 are suppressed, RIPK1 and RIPK3 assemble into a high molecular weight intracellular platform termed the necrosome [23,24]. Recruitment of MLKL to the necrosome prompts RIPK3-mediated phosphorylation of Ser345 in mouse MLKL and Thr357/Ser358 in human MLKL [25–27]. Phosphorylated MLKL undergoes a conformational change allowing disengagement from the necrosome and the assembly of pro-necroptotic oligomers [28–31] which traffic to the plasma membrane, where a critical threshold is exceeded to enable membrane rupture and cell death. Inflammatory mediators released during necroptosis promote further cell death and inflammation in a process termed ‘necroinflammation’ [32,33].

Several mouse studies have evaluated the role of necroptosis in kidney IRI using either genetic ablation or pharmacological inhibition. *Ripk3*-deficient (*Ripk3*-ko) mice were protected from IRI [34,35], as were *Mlkl* knockout (*Mlkl*-ko) mice [36,37]. While *Ripk1* deletion is not tolerated in mice owing to embryonic lethality, inhibition of RIPK1 with Necrostatin-1 (Nec-1) [38] was also protective [39]. However, Nec-1 is not specific for RIPK1 as it also inhibits indoleamine 2,3-dioxygenase (IDO), which may influence the immune response [40–42]. Furthermore, Nec-1 is toxic [42] and is reported to block another cell death pathway, ferroptosis [43]. Nec-1s has been developed more recently as a stable version of Nec-1 that lacks IDO-inhibitory activity, is less toxic and does not act as a ferrostatin [44]. Nec-1s locks RIPK1 in an inactive conformation [45], preventing its phosphorylation and ubiquitination within the necrosome [46]. Nec-1s treatment protected against TNF-induced systemic inflammatory response syndrome [47] and folic acid-induced AKI [48], but its effect in kidney IRI has not yet been assessed. The RIPK3 inhibitor, GSK´872 [49,50], which binds and inhibits catalytic activity of the kinase domain of RIPK3 [49,51], has also shown protection in several mouse models, including acute lung injury [52] and lupus nephritis [53]. There has been one report that GSK´872 protects against kidney IRI in mice, although the degree of protection was modest [54], warranting further investigation.

Components of the necroptosis pathway, RIPK1 and RIPK3, have also been implicated in the pathogenesis of kidney fibrosis following AKI. Studies in mice using the unilateral ureteric obstruction (UUO) and folic acid models suggest that RIPK1 and RIPK3 contribute to kidney fibrosis [55–57]. *Ripk3*-ko but not *Mlkl*-ko mice were protected from kidney fibrosis in UUO, pointing to the involvement of additional mechanisms beyond necroptosis [51]. Furthermore, inhibition of RIPK1 with daily Nec-1 treatment attenuated kidney fibrosis in the UUO model, although it remains unclear whether this could be attributed to RIPK1 inhibition or to off-target effects of IDO inhibition [55].

In the present study, we evaluated for the first time the effect of the RIPK1 inhibitor, Nec-1s, in a model of IR-induced AKI and compared the impact of RIPK3 inhibition with GSK´872. We also examined whether treatment with these inhibitors commencing after the peak of IR-induced AKI could attenuate the subsequent development of kidney fibrosis. We demonstrate that Nec-1s protects against AKI and that both Nec-1s and GSK´872 reduce kidney fibrosis following ischemia-reperfusion in a mouse model.

## Results

### Prophylactic treatment with Nec-1s, but not GSK´872, protects against kidney IRI

**Figure 1:**
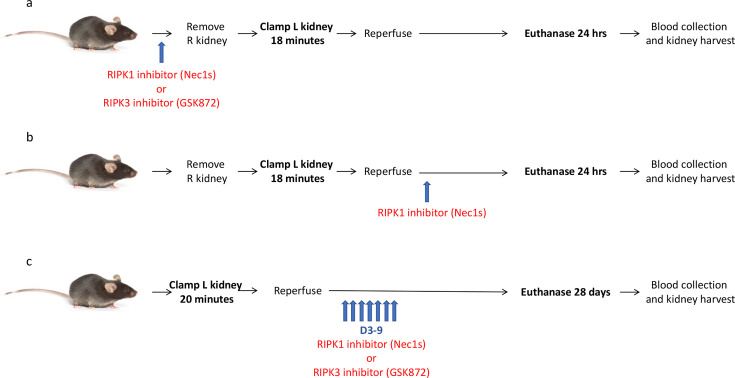
Mouse models of unilateral kidney ischemia-reperfusion injury. (**a**) Model of acute kidney injury (AKI) following ischemia-reperfusion. Nec-1s, GSK´872 or a vehicle control were injected 30 min prior to ischemia, with sample collection at 24 hr. (**b**) Model of AKI in which Nec-1s was administered 15 min after reperfusion. (**c**) Model of chronic kidney injury following IR. Mice received Nec-1s, GSK´872 or a vehicle control daily on days 3–9 after IR, with sample collection at day 28. R, right; L, left.

**Figure 2:**
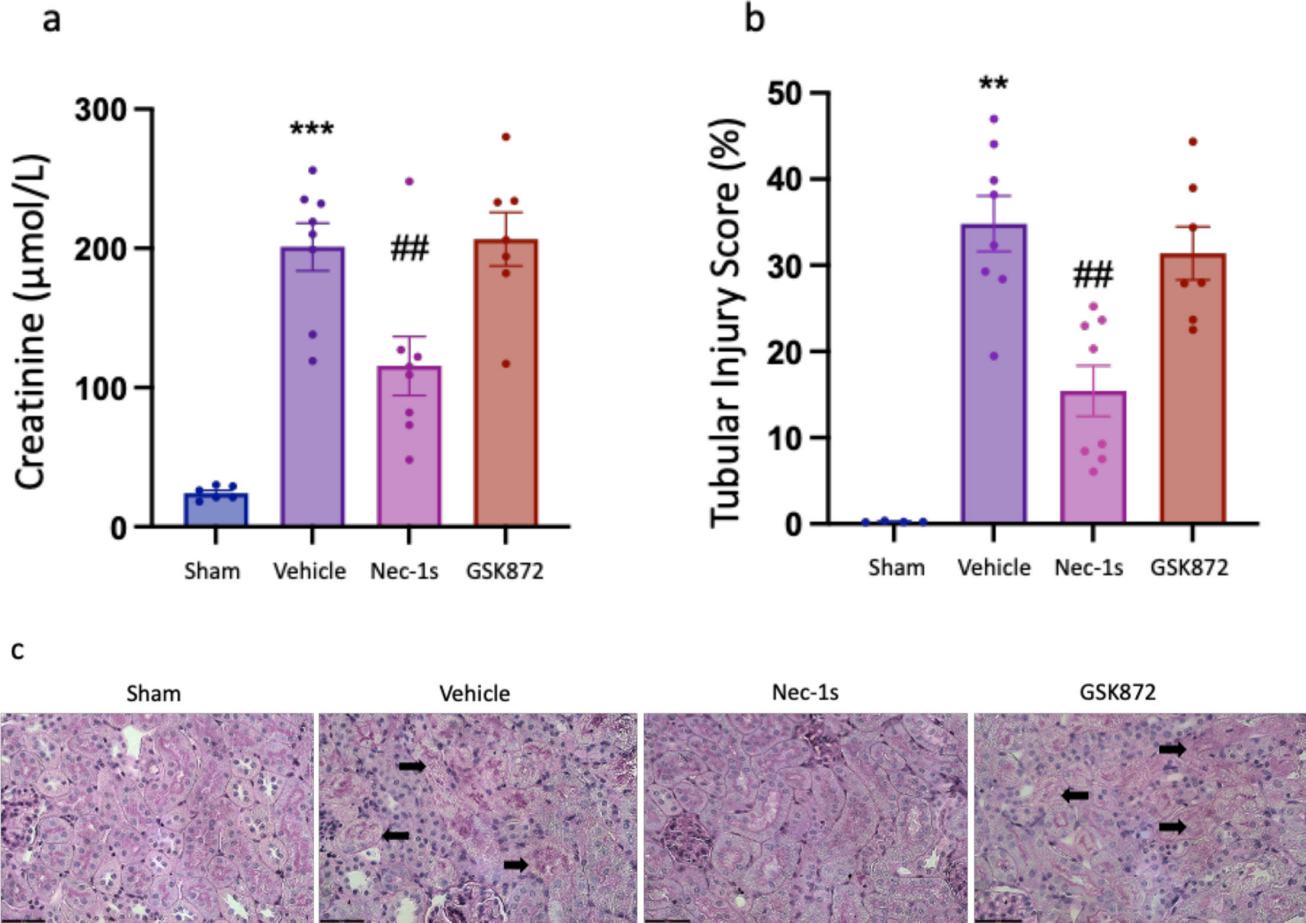
Prophylactic treatment with Nec-1s, but not GSK´872, protects against kidney ischemia reperfusion injury at 24 hr. (**a**) Serum creatinine; (**b**) tubular injury score; (**c**) representative PAS-stained kidney sections, with arrows indicating necrotic tubules (image obtained using Olympus UPlanApo 40× objective lens; 0,85 N.A. Scale bar 50 μm). **P<0.01, ***P<0.001 compared with sham procedure; ##*P*<0.01 compared with vehicle treatment. Data presented as mean ± SEM. *N*=4–8 per group. PAS, periodic acid-Schiff.

**Figure 3:**
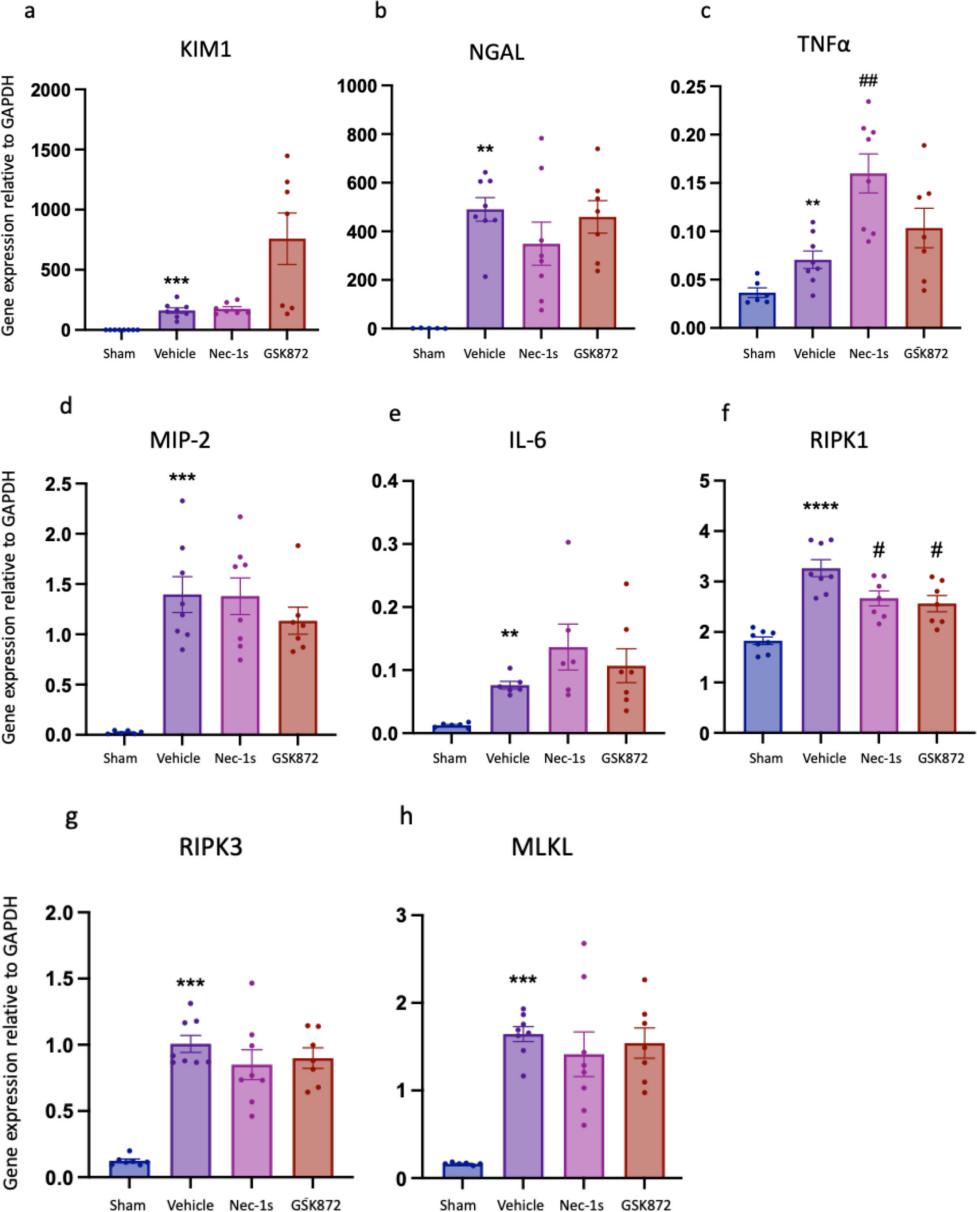
Up-regulation of gene expression of kidney injury marker, pro-inflammatory and necroptosis pathway components at 24 hr after kidney IR is unaffected by prophylactic treatment with Nec-1s or GSK´872. (**a**) *Kim1*; (**b**) *Ngal*; (**c**) *Tnfa*; (**d**) *Mip2*; (**e**) *Il6*; (**f**) *Ripk1*; (**g**) *Ripk3*; (**h**) *Mlkl*. **P<0.01, ***P<0.001, ****P<0.0001 compared with sham procedure; #*P*<0.05, ##*P*<0.01 compared with vehicle treatment. Data presented as mean ± SEM . *n*=6–8 per group.

Wild-type (WT) mice were treated intraperitoneally (i.p.) with either 6 mg/kg Nec-1s, 5 mg/kg GSK´872 or a 10% DMSO vehicle control 30 min prior to warm unilateral kidney ischemia (Figure 1a). Drug doses and administration were based on safety and efficacy data from other murine models [47,52]. Readouts at 24 hr included kidney function (serum creatinine), morphological injury (blinded assessment of Tubular Injury Scores) and gene expression of molecules relevant to necroptosis (*Ripk1, Ripk3, Mlkl*) and inflammation (*Tnfa, Mip2, Il6, Il33*). Compared with Sham mice, vehicle-treated IRI mice showed significant kidney injury and dysfunction. Vehicle-treated IRI mice had increased serum creatinine (*P*=0.0007) (Figure 2a), increased histological evidence of tubular injury (*P*=0.0040) (Figure 2b and c) and up-regulated gene expression of kidney injury markers (*Kim1*: *P*=0.0002; *Ngal: P*=0.0016), pro-inflammatory molecules (*Tnfa: P*=0.0080; *Mip2: P*=0.0007; *Il6*: *P*=0.0022) and necroptosis pathway components (*Ripk1*: *P*=0.0002; *Ripk3*: *P*=0.0003; *Mlkl*: *P*=0.0007) (Figure 3). RIPK1 inhibition with Nec-1s, but not RIPK3 inhibition with GSK´872, reduced kidney injury, with significantly reduced serum creatinine (*P*=0.0045) and tubular injury score (*P*=0.0056) compared with vehicle-treated mice (Figure 2). There was no difference in the gene expression of kidney injury markers *Kim1* or *Ngal* at this timepoint (Figure 3a and b). Interestingly, Nec-1s increased *Tnfa* gene expression however, there was no difference in *Mip2* or *Il6* mRNA levels between drug and vehicle groups (Figure 3c–e). Both Nec-1s and GSK´872 reduced expression of *Ripk1*, with no effect on *Ripk3* or *Mlkl* expression (Figure 3f–h).

### Delaying Nec-1s treatment until after reperfusion does not abrogate protection

**Figure 4:**
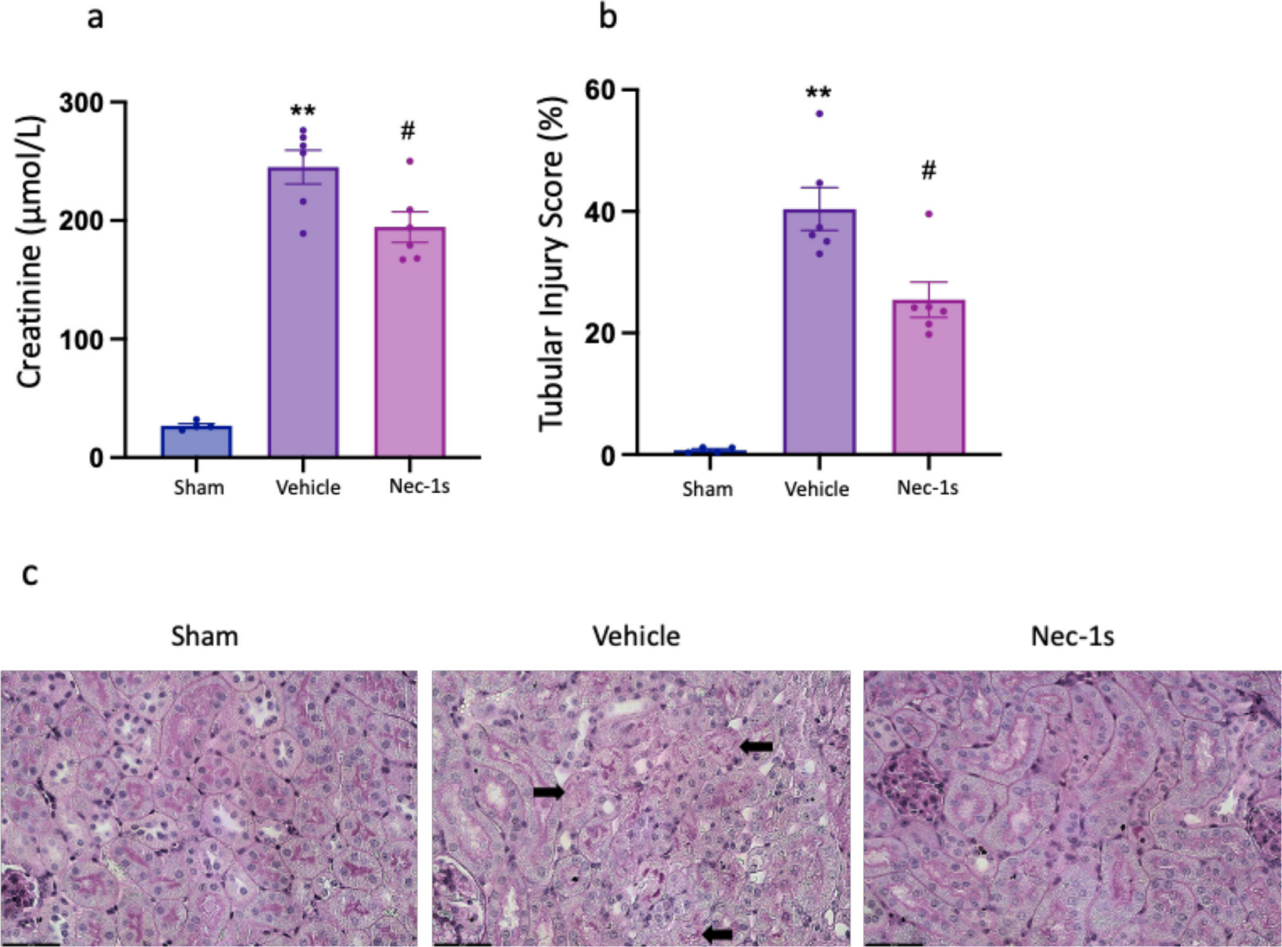
Therapeutic treatment with Nec-1s after ischemia-reperfusion reduces kidney injury at 24 hr. (**a**) Serum creatinine; (**b**) tubular injury score; (**c**) representative PAS-stained kidney sections following vehicle or Nec-1s treatment with arrows indicating necrotic tubules (image obtained using Olympus UPlanApo 40× objective lens; 0,85 N.A. Scale bar 50 μm). *P 0.05, **P<0.01 compared with sham procedure; #*P*<0.05 compared with vehicle treatment. Data presented as mean ± SEM. *n=*4–6 per group. PAS, periodic acid-Schiff.

**Figure 5:**
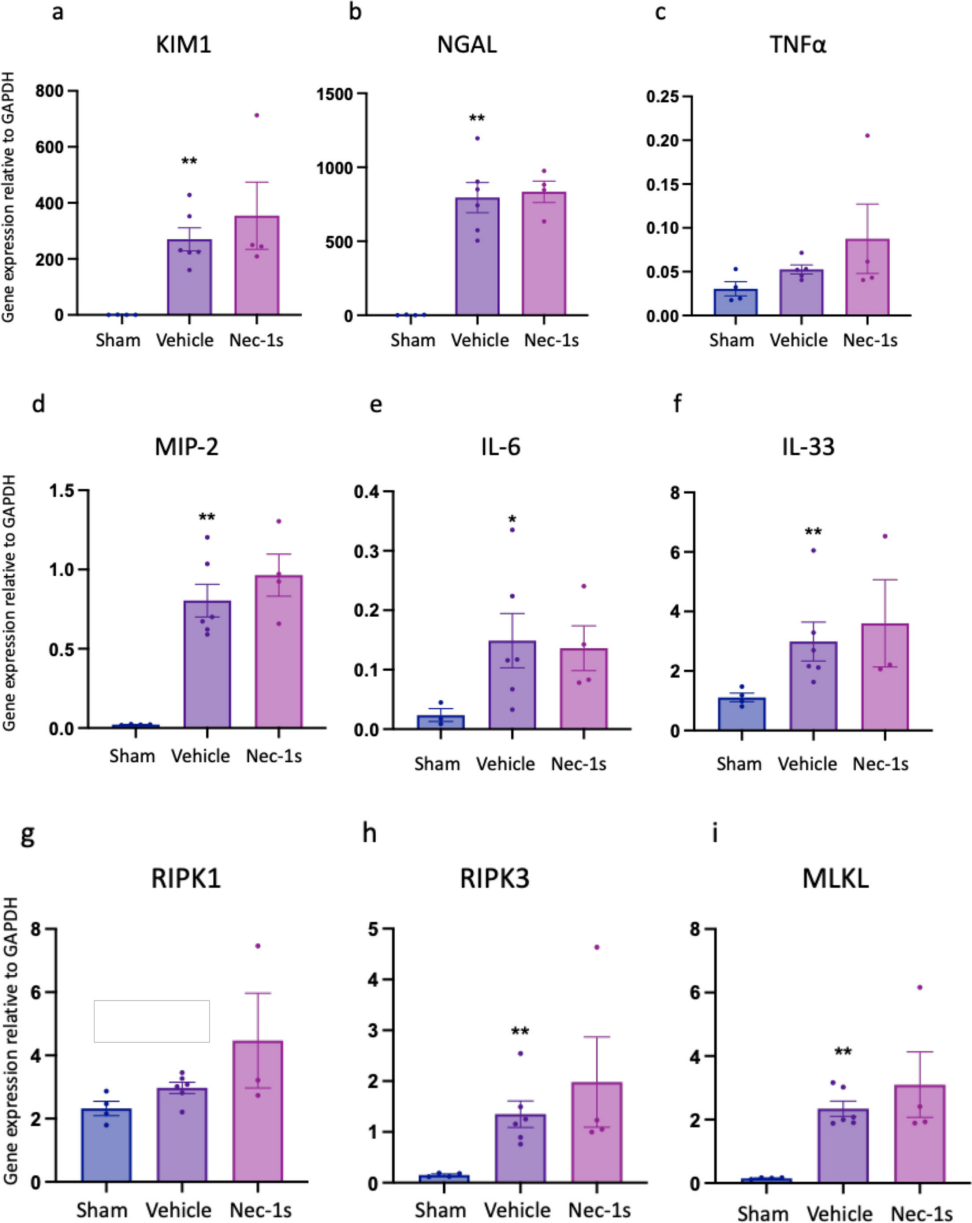
Therapeutic treatment with Nec-1s does not affect gene expression of kidney injury, pro-inflammatory or necroptosis pathway genes at 24 hr following reperfusion. (**a**) *Kim1*; (**b**) *Ngal*; (**c**) *Tnfa*; (**d**) *Mip2*; (**e**) *Il6*; (**f**) *IL33*; (**g**) *Ripk1*; (**h**) *Ripk3*; (**i**) *Mlkl*. *P<0.05, **P<0.01 compared with sham procedure. Data presented as mean ± SEM. *n*=4–6 per group.

As prophylactic treatment with Nec-1s 30 min prior to ischemia reduced kidney injury, we next tested whether administration in a more therapeutically relevant setting – post-injury – conferred protection (Figure 1b). WT mice were treated i.p. with 6 mg/kg Nec-1s or a vehicle control 15 min after reperfusion. Therapeutic treatment with Nec-1s reduced serum creatinine (*P*=0.0260) and tubular injury score (*P*=0.0260) at 24 hr (Figure 4), although there was no difference in the expression of kidney injury markers, pro-inflammatory or necroptosis pathway genes at this timepoint (Figure 5). Prophylactic GSK´872 treatment did not protect against AKI and, therefore, was not tested post reperfusion.

### Treatment with either Nec-1s or GSK´872 after the peak of AKI reduces kidney fibrosis

**Figure 6:**
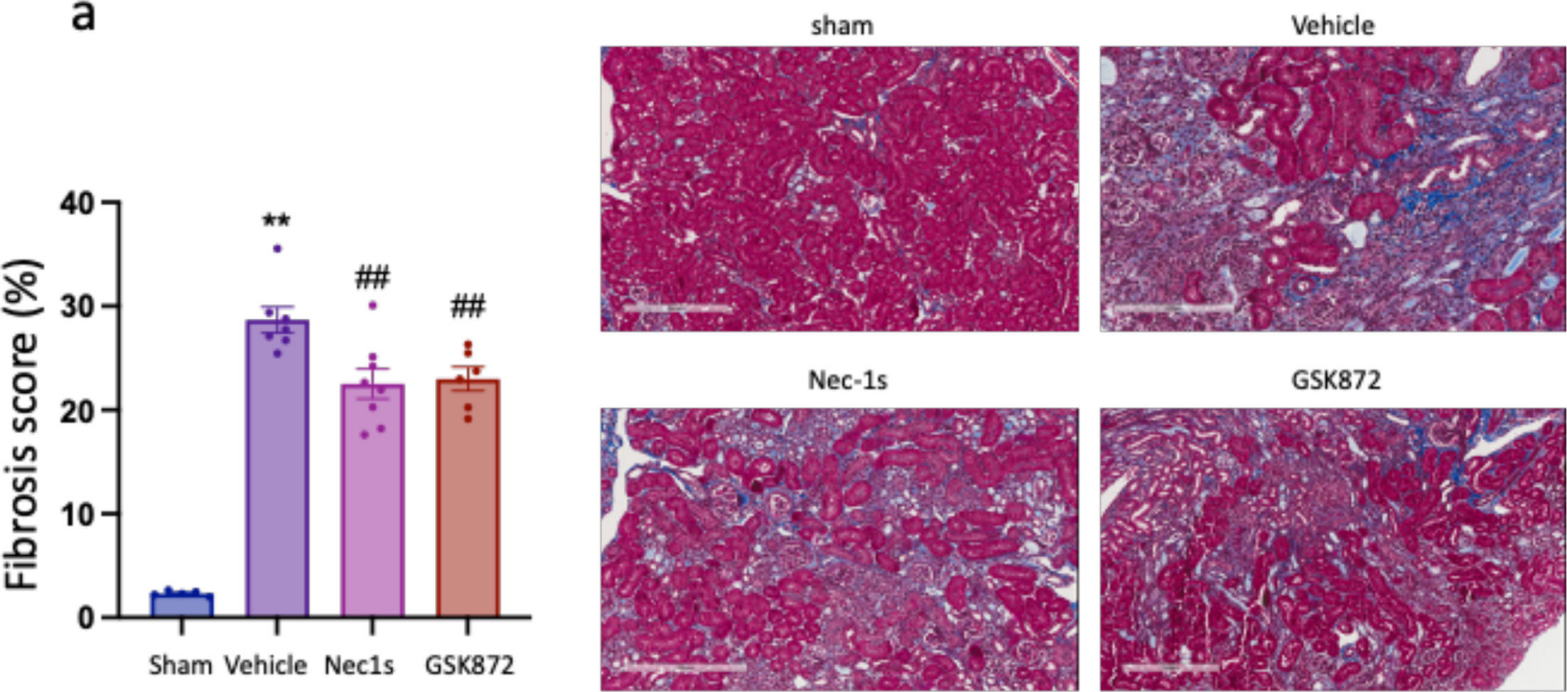
Inhibition of RIPK1 or RIPK3 after IRI is established reduces kidney fibrosis at day 28. (a) Kidney fibrosis score at 28 days with representative images of Mason Trichrome staining in each group (image obtained using Olympus UPlanApo 20× objective lens. Scale bar 20 μm). **P<0.01 compared with sham procedure; ##*P*<0.01 compared with vehicle treatment. Data presented as mean ± SEM. *n*=5–8 per group.

**Figure 7:**
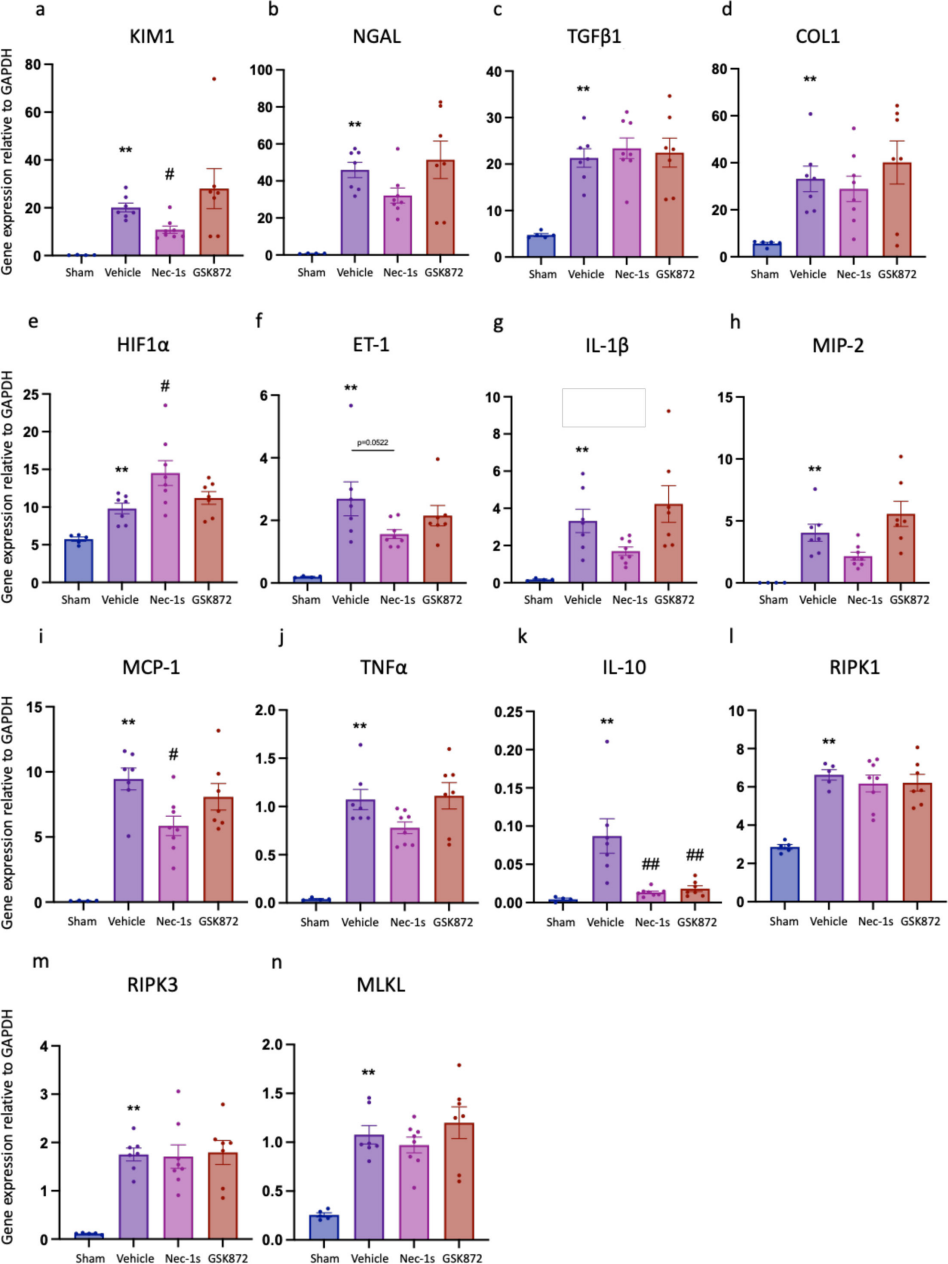
The expression of kidney injury, fibrosis, pro inflammatory and necroptosis pathway genes is up-regulated at day28 post-reperfusion. (**a**) *Kim1*; (**b**) *Ngal*; (**c**) Tgfb; (**d**) Col1; (**e**) *HIF1a*; (**f**) *Et1*; (**g**) *Il1b*; (**h**) *Mip2*; (**i**) *Mcp1*; (**j**) *Tnfa*; (**k**) *Il10*; (**l**) *Ripk1*; (**m**) *Ripk3*; (**n**) *Mlkl*. **P<0.01 compared with sham procedure; #*P*<0.05, ##*P*<0.01 compared with vehicle treatment. Data presented as mean ± SEM. *n*=5–8 per group.

To test the effect of inhibitor treatment after the peak of ischemic AKI on the development of fibrosis, we used a model in which the left kidney was subjected to ischemia-reperfusion, whereas the right kidney remained intact, with assessment of fibrosis at day 28 (Figure 1c). Mice received 1.65 mg/kg/day Nec-1s, 1 mg/kg/day GSK´872 or a vehicle control from days 3 to 9 post-IRI. Cumulative drug doses were based on safety data from other murine models. The starting point of treatment was based on our finding that AKI in this model has peaked and is resolving by day 3 following IR [37]. Vehicle-treated IRI mice showed significant left kidney fibrosis at day 28 versus Sham mice (*P*=0.0025) (Figure 6a), accompanied by increased expression of the pro-fibrotic genes *Tgfb* (*P*=0.0025), *Col1* (*P*=0.0025)*, Hif1a* (*P*=0.0025) and *Et1* (*P*=0.0061) (Figure 7c and f). Gene expression of the kidney injury markers *Kim1* (*P*=0.0061) and *Ngal* (*P*=0.0061) was modestly elevated in the vehicle group versus the Sham group (Figure 7a and b), indicating ongoing injury to the left kidney. Several pro-inflammatory genes, including *Tnfa* (*P*=0.0061), *Il1b* (*P=0.0061*), *Mip2* (*P*=0.0061) and *Mcp1* (*P*=0.0061), were also up-regulated in the vehicle group, as were *Ripk1* (*P*=0.0079), *Ripk3* (*P*=0.0025) and *Mlkl* (*P*=0.0025) (Figure 7).

Mice receiving Nec-1s or GSK´872 treatment had reduced left kidney fibrosis scores at day 28 compared with vehicle-treated mice (Nec-1s: *P*=0.0011; GSK´872: *P*=0.0038) (Figure 6a). Neither treatment affected kidney expression of *Tgfb* or *Col1* (Figure 7c and d); however, Nec-1s treatment increased *Hif1a* and reduced *Et1* gene expression (Figure 7e and f) at this timepoint. Nec-1s, but not GSK´872, reduced expression of *Kim1* and *Mcp1* (Figure 7a and i). There was no difference in core necroptosis pathway effector gene expression between drug- and vehicle-treated mice (Figure 7l and n).

## Discussion

There is increasing evidence that necroptosis contributes to IRI of solid organs including the liver, brain, heart and kidney [58], in addition to barrier tissues, such as the gut, skin and lung epithelium [59–62]. The core function of the terminal effector of the necroptosis pathway, MLKL, is in executing necroptotic cell death; therefore, MLKL offers the most specific target to block necroptotic cell death and reduce kidney injury. However, no specific inhibitors of mouse MLKL are available [63], restricting pharmacological targeting in mouse models to RIPK1 and RIPK3. Previous mouse IRI studies have used Nec-1 to inhibit RIPK1, but this inhibitor has several drawbacks including toxicity and limited specificity, as it also inhibits indoleamine 2,3-dioxygenase (IDO) activity and ferroptosis. We used Nec-1s, a more specific and less toxic derivative of Nec-1, to confirm that prophylactic RIPK1 inhibition protects against kidney IRI in mice. Furthermore, we demonstrated that protection was observed even when Nec-1s was administered 15 min *after* reperfusion. This observation is consistent with our finding that the period 3–12 hr after reperfusion is a critical phase for the activation of necroptosis in kidney proximal tubular cells [37] and may have important implications for clinical translation. Whilst IRI following kidney transplantation may be anticipated and thus potentially treated prophylactically, the vast majority of kidney IRI in clinical settings remains unpredictable. The protection afforded by therapeutic Nec-1s treatment suggests that clinical application of necroptosis inhibitors may be extended to critically unwell patients requiring intensive care support, patients with significant hypotension, or following major vascular surgery such as repair of aortic aneurysm rupture resulting in IRI. In the transplant setting, necroptosis inhibitors could be added to the perfusate fluid during kidney normothermic machine perfusion to reduce the anticipated IRI, circumventing the ethical issues of treating a deceased donor and mitigating concerns regarding off-target treatment effects in a transplant recipient.

Unlike Nec-1s, the RIPK3 inhibitor GSK´872 was not protective when administered prophylactically, at a dose (5 mg/kg) that was effective in a mouse model of acute lung injury [52]. GSK´872 has been reported to provide modest protection against kidney IRI in mice [54], although model differences (e.g. higher dose (10 mg/kg), younger mice, bilateral ischemia) may partly account for this discrepancy. There is evidence that GSK´872 at high doses promotes switching from RIPK3-dependent necroptosis to Caspase-8-dependent apoptosis *in vitro* [49] and *in vivo* [64], as well as induces caspase-dependent cytotoxicity [49,63]. In this light, future studies of two recently identified more potent RIPK3 inhibitors, UH15-38 [65] and AZD5423 [54], in a kidney IRI model hold great interest.

IRI is a common cause of AKI, which may progress to chronic kidney disease. There are no available clinical therapies with proven efficacy in preventing this progression. Previous mouse studies have examined the role of RIPK1 and RIPK3 in kidney fibrosis using the UUO and folic acid models [55–57]. These studies, using Dabrafenib, Fluorofenidone or Nec-1, commenced treatment at the time of kidney injury. It was, therefore, unclear whether the reduction in kidney fibrosis observed was owing to reduced AKI resulting from less necroptotic cell death, or to inhibition of necroptosis-independent roles of RIPK1 and RIPK3 in fibrogenesis. Our experimental protocol addressed this issue by commencing drug treatment after AKI was already established [37]. We showed that inhibiting either RIPK1 with Nec-1s or RIPK3 with GSK´872 from days 3–9 attenuated kidney fibrosis at day 28. This, together with previous findings that *Mlkl*-ko mice were not protected from kidney fibrosis [37,51], suggests that RIPK1 and RIPK3 indeed play a role in kidney fibrosis that is independent of necroptotic cell death.

Nec-1s and GSK´872 reduced but did not eliminate kidney fibrosis. Our data do not establish whether transiently inhibiting RIPK1 or RIPK3 early during the AKI to chronic kidney disease transition reduced the severity of fibrosis or merely delayed its onset. The fact that profibrotic gene expression at day 28 was similarly elevated in treated and untreated mice (Figure 6) suggests the latter may be the case and can be confirmed by extending the analysis time in future studies. It is, therefore, conceivable that continuing treatment for longer periods may provide a greater reduction in fibrosis. Furthermore, given that prophylactic or therapeutic Nec-1s treatment afforded protection in the AKI model, treatment at the time of IR and continuation for a prolonged period may more effectively prevent kidney fibrosis by targeting both necroptotic AKI and fibrogenesis. Combining RIPK1 and RIPK3 inhibition may provide additive benefit, and further drug treatment studies are warranted.

The molecular mechanisms by which RIPK1 and RIPK3 contribute to kidney fibrosis during the transition from AKI to chronic kidney disease are the subject of ongoing investigation. RIPK1 and RIPK3 may promote fibrosis following IRI via inflammatory pathways, both can up-regulate inflammatory gene expression [66], promote cytokine production [66–68] and direct interferon-β synthesis induced by lipopolysaccharide [69]. Furthermore, existing literature suggests that RIPK3 can promote fibrosis via the protein kinase B (AKT)-dependent activation of ATP citrate lyase pathway, with downstream activation of mammalian target of rapamycin (mTOR) pathways [56,70–72]. mTOR contributes to kidney fibrosis by increasing epithelial-to-mesenchymal transition (EMT), stimulating fibroblast proliferation and M2 macrophage polarisation [73].

Whilst the present study primarily focuses on the role of necroptosis in kidney IRI, we and other groups have demonstrated a relationship between various cell-death pathways such that resistance to one pathway may sensitise cells to death via another pathway [36,37]. In particular, the role of ferroptosis in kidney IRI is emerging [74]. Future studies would benefit from additional analysis including the evaluation of lipid peroxidation after RIPK1 or RIPK3 inhibition to delineate the various forms of cell death crosstalk.

In summary, we demonstrate that treatments targeting components of the necroptosis pathway reduce both AKI and fibrosis following ischemia-reperfusion in a mouse model. Necroptosis inhibitors are currently in clinical development, with a phase 2 trial currently evaluating the RIPK1 inhibitor SAR443122 in patients with cutaneous lupus erythematosus (NCT04781816), whilst recent published studies evaluating another RIPK1 inhibitor, GSK2982772, did not show efficacy in patients with moderate to severe rheumatoid arthritis [75] or with active ulcerative colitis [76]. Our results support extending clinical trials to patients with kidney IRI to reduce kidney injury and improve patient outcomes.

## Materials and methods

### Animal and ethical statement

C57BL/6J WT mice were sourced from the Animal Research Centre (ARC, Perth, WA, Australia). Mice were housed in microisolator cages in a pathogen-free facility with a 12 hr light-dark cycle under standard conditions of temperature and humidity and fed commercial mouse chow diet with free access to drinking water. All animal experiments were conducted in compliance with the Australian Code of Practice for the Care and Use of Laboratory Animals for Scientific Purposes (Eighth edition, 2013), the Prevention of Cruelty to Animals Act 1986, Victoria, Australia. Authors complied with the ARRIVE guidelines (V2.0). All experiments were approved by the Animal Ethics Committee of St. Vincent’s Hospital Melbourne (SVHM AEC protocols 026/17 and 022/20).

### Acute kidney ischemia-reperfusion injury model

The basic schema of the kidney IRI model is shown in Figure 1a and b. Ten-to-twelve-week-old male mice were anaesthetised by intraperitoneal injection of ketamine (100 mg/kg) and xylazine (15 mg/kg), followed by right nephrectomy prior to left renal pedicle clamping for 18 min using a microvascular clamp (Roboz, Rockville, MD, U.S.A.). After the removal of the clamp, the kidney was assessed for even reperfusion prior to abdominal wound closure. Mice received 200 µL warmed (37°C) normal saline i.p. postoperatively, and core body temperature was maintained at 35.5–36.5°C throughout. Sham mice had a right nephrectomy, but the left renal pedicle was not clamped. Mice were euthanised by ketamine/xylazine overdose and exsanguination at 24 hr after reperfusion. Blood and kidney samples were obtained to assess kidney function, kidney injury, inflammation and necroptosis.

### Kidney fibrosis following the IRI model

This model was similar to the acute kidney IRI model, except that a right nephrectomy was not performed, the left kidney was clamped for 20 min and mice were euthanised 28 days after IR (Figure 1c).

### Inhibitor treatments

*Acute kidney IRI model:* 6 mg/kg Nec-1s (EZSolution^TM^ Nec-1s, BioVision #2535–5, Milpitas, CA, U.S.A.), 5 mg/kg GSK´872 (EZSolutionTM GSK´872, BioVision #B1501-5) or a 10% DMSO vehicle control were injected i.p. 30 min before ischemia (prophylactic treatment) (Figure 1a) or 15 min after reperfusion (therapeutic treatment) (Figure 1b). Drug doses were based on safety and efficacy data from other murine models [77]. Kidney fibrosis following the IRI model: 1.65 mg/kg Nec-1s, 1 mg/kg GSK´872 or a 10% DMSO vehicle control were administered i.p. daily on days 3–9 following IR (Figure 1c).

### Analysis of kidney function

Serum creatinine was measured using a kinetic colorimetric assay on a COBAS Integra 400 Plus analyser (Roche, Castle Hill, NSW, Australia).

### Assessment of tubular injury score

Kidney tissue blocks were fixed overnight in 10% formalin and embedded in paraffin wax. Four-micrometre sections were cut and de-waxed prior to periodic acid-Schiff staining [78]. Each section was divided into 12 regions. A representative area of each region was viewed under 400× magnification, with a focus on the corticomedullary junction. A score was derived by calculating the number of damaged proximal tubules as a percentage of total proximal tubules manually counted in each area [79]. Markers of a damaged tubule included signs of tubular atrophy, tubular dilatation, tubular cast formation, vacuolisation and tubular cell degeneration with loss of brush border or thickening of tubular basement membranes. An average of the 12 scores obtained per section was calculated. Scoring was performed under blinded conditions by personnel trained by an experienced veterinarian pathologist.

### Assessment of kidney fibrosis

Four-micrometre paraffin sections were de-waxed and placed in Bouin’s fixative overnight prior to Masson’s Trichrome staining. Stained sections were scanned using an Aperio ScanScope (Leica Biosystems, North Ryde, NSW, Australia) to generate a digitised image of the whole section. Fibrosis was quantified using the positive pixel algorithm to detect areas of collagen-positive tissue and expressed as a ‘positivity score’ [79,80].

### Reverse transcription-quantitative PCR

Harvested kidneys were stored in RNA Later^®^ (Sigma-Aldrich Pty. Ltd, Australia) at 4°C for 24–48 hr, followed by storage at −80°C until processing. Total RNA was extracted using the ReliaPrep™ RNA Tissue Miniprep system (Promega Australia, Alexandria, NSW, Australia). RNA concentration and quality were measured using a Fluorostar Omega multimode microplate reader (BMG Labtech, Mornington, Australia). First-strand complementary DNA (cDNA) was generated in a reaction volume of 22 µL containing 1 µg oligo (dT), 1 µg random hexamers (Invitrogen, Carlsbad, CA, U.S.A.), 12 µg of RNA and sterile MQ-H_2_O. The reaction was incubated for 10 min at 70°C. Following this, a 28 µL mix comprising 2.5 µL 10 mM dNTPs, 1 µL SuperScript III recombinant reverse transcriptase, 1 µL RNaseOUT recombinant ribonuclease inhibitors, 2.5 µL 0.1 M DTT, 10 µL 5× first-strand buffer (Invitrogen, Carlsbad, CA, U.S.A.) and 11 µL MQ-H_2_O was added to the first reaction. Reverse transcription was performed at 42°C for 1 hr and 70°C for 10 min. The cDNA was stored at −20°C. Quantitative real-time PCR was performed using the TaqMan Universal PCR Master Mix system and TaqMan Gene Expression Assays (Applied Biosystems, Carlsbad, CA, U.S.A.), using a 7400Fast Real-Time PCR System (Applied Biosystems). Quantified genes are listed in Table S1.

### Statistical analysis

Results were expressed as mean ± SEM unless otherwise indicated. A Mann–Whitney U-test was used to assess significance of injury (sham vs. isotype control). When determining the effect of treatments, a Kruskal–Wallis test was used for multiple treatments whilst a Mann–Whitney U-test was used to compare one treatment with a vehicle control. Statistical analyses were performed using GraphPad Prism version 9.3 (GraphPad, San Diego, CA, U.S.A.) with *P* <0.05 considered significantly.

## Supplementary material

online supplementary table 1.

## Data Availability

The raw data supporting the conclusions of this article will be made available by the authors, without undue reservation.

## Abbreviations

AKI: acute kidney injury
IRI: ischemia-reperfusion injury
MLKL: mixed lineage kinase domain-like
Nec-1: Necrostatin-1
UUO: unilateral ureteric obstruction
WT: Wild-type
i.p.: intraperitoneally
