# Supplementary material for: Inhibition of RIPK1 or RIPK3 kinase activity post ischemia-reperfusion reduces the development of chronic kidney injury
Source: Biochem J. 2025 Jan 22;482(2):BCJ20240569. doi: 10.1042/BCJ20240569 (PMC12220529; doi:10.1042/BCJ20240569)
Supplement: online supplementary table 1. [file bcj-482-2-BCJ20240569-s001.pdf]

Suppl Table 1: TaqMan gene expression assays

Gene name	Gene Symbol	Assay ID
Kidney injury molecule 1	*Kim1*	Mm00506686_m1
Neutrophil gelatinase-associated lipocalin	*Ngal*	Mm01324470_m1
Interleukin 1 beta	*Il1b*	Mm00434228_m1
Macrophage inflammatory protein-2	*Mip2*	Mm00436450_m1
Receptor interacting protein kinase 1	*Ripk1*	Mm00436354_m1
Receptor interacting protein kinase 3	*Ripk3*	Mm00444947_m1
Mixed lineage kinase domain-like protein	*Mlkl*	Mm01244222_m1
Interleukin 6	*Il6*	Mm00446190_m1
Interleukin 33	*Il33*	Mm00505403_m1
Tumour necrosis factor alpha	*Tnfa*	Mm00443258_m1
Transforming growth factor beta 1	*Tgfb1*	Mm01178820_m1
Collagen type 1	*Col1*	Mm00483888_m1
Collagen type 4	*Col4*	Mm00802386_m1
Hypoxia inducible factor 1 subunit alpha	*Hif1a*	Mm00468869_m1
Monocyte chemoattractant protein 1	*Mcp1*	Mm00441242_m1
Endothelin 1	*Et1*	Mm00438656_m1
Interleukin 10	*Il10*	Mm01288386_m1
Glyceraldehyde 3-phosphate dehydrogenase	*Gapdh*	Mm99999915_g1
